# New Onset Tinnitus after High-Frequency Spinal Cord Stimulator Implantation

**DOI:** 10.1155/2019/5039646

**Published:** 2019-05-02

**Authors:** Alexander V. Golovlev, Michael G. Hillegass

**Affiliations:** Department of Anesthesiology and Perioperative Medicine, Medical University of South Carolina, Charleston, South Carolina, USA

## Abstract

The most common complications of spinal cord stimulation (SCS) therapy are generally related to surgical site infection and hardware malfunction. Less well understood are the adverse neurological effects of this therapy. We present the case of a patient who underwent placement of a Senza HF10 high-frequency spinal cord stimulator with subsequent development of tinnitus, vertigo, intermittent involuntary left facial twitches, and perioral numbness. These symptoms resolved following deactivation of her device. To further explore these less common neurologic complications of SCS therapy, a review of literature and a review of the U.S. Food and Drug Administration Manufacturer and User Facility Device Experience database are included. Further research and investigation in this area are needed so that clinicians and patients may have more complete knowledge and understanding of the potential treatment-limiting complications of spinal cord stimulation.

## 1. Introduction

Spinal cord stimulation is a proven treatment modality for a variety of pain pathologies. The first clinical report of successful use of spinal cord stimulation (SCS) was described in 1967 by Shealy et al. [1] using a single epidural lead programmed at 10-50 Hz tonic frequency. Since then, the technology of spinal cord stimulation has advanced at an accelerated pace. Spinal cord stimulator technology until 2015 had relied on low frequency electrical stimulation in the 50-120 Hz range with either constant-current or constant-voltage waveforms to achieve paresthesia for pain relief [2]. Recently, new classes of “paresthesia-free” waveforms have demonstrated long-term durable pain relief. These include burst stimulation [3], high pulse density, and high frequency stimulation [4, 5]. Specifically, high frequency stimulation relies on a continuous waveform delivered at 10 kHz [4]. The first-in-class high frequency spinal cord stimulator to gain FDA approval was the Senza HF10 device from Nevro Corporation (Redwood City, CA) [6].

The precise mechanism of spinal cord stimulation has not been fully elucidated [2, 7]. Moreover, adverse neurologic effects of this therapy are not well known or understood. In this case report, we present a patient who experienced persistent and disabling tinnitus, vertigo, and perioral numbness with intermittent nausea, vomiting, and diarrhea status after high frequency spinal cord stimulator placement and activation that resolved with deactivation of the device.

## 2. Case Presentation

**Figure 1:**
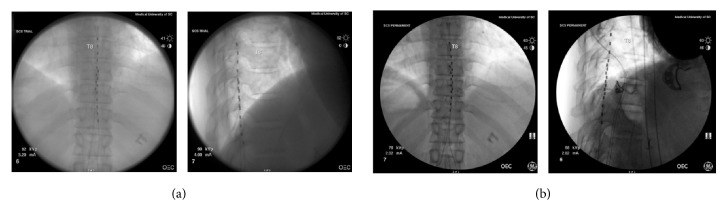
AP and lateral fluoroscopic images demonstrating location of epidural placement of trial leads (a) and permanent leads (b).

The patient is a 50-year-old female who had a 4-year history of left lumbar radiculopathy which was precipitated from a lifting injury that did not resolve following L4-5 microdiscectomy. She had previously been unsuccessfully managed with epidural steroid injections and multimodal analgesics. A nerve conduction study was unremarkable for any pathology. She was diagnosed with failed back surgery syndrome and counseled regarding her treatment options, including spinal cord stimulation. She elected to pursue spinal cord stimulation with the Nevro Senza HF10 system. As part of her evaluation for SCS placement, the patient was seen at the behavioral medicine clinic and underwent presurgical psychological testing and evaluation by a board-certified psychologist. Her psychological assessment profile suggested a high likelihood of a good postoperative outcome. She had a history of depression and anxiety, both of which were stable and under medical management. The patient underwent an uneventful SCS trial with near 100% relief of her back and leg pain and wished to proceed with permanent implant. Her permanent placement procedure was uneventful with leads placed at the same levels as her trial leads (Figure 1.) with the tip of the right lead at the top of the T8 vertebral body and left lead tip at the mid T9 vertebral body. The device was activated in the post-anesthesia care unit with bipole setting on electrodes 10 and 11.

The first day following her procedure, the patient noted a buzzing or a chirping sound predominately in her left ear as well as vertigo, intermittent involuntary left facial twitches, and perioral numbness, all of which were new to her. She also reported nausea, vomiting, and diarrhea, which she has attributed to anxiety in the past. She was subsequently evaluated by ENT around 4 weeks postoperatively and underwent an audiological evaluation with no abnormal findings. There was concern that the tinnitus could be related to her stimulator. She also later complained of implanted pulse generator (IPG) site pain and stinging sensations. Attempts at reprograming her SCS system with a bipole settings on leads 4 and 5 failed to resolve her ongoing neurologic symptoms. The patient was subsequently advised to deactivate her system to see if her symptoms would improve. Two days after deactivation of her system, the patient reported complete resolution of her symptoms. She was again evaluated and spine radiographs were performed which demonstrated unchanged position of her SCS leads. She was not interested in reactivating her system to assess for return of symptoms. The SCS system was subsequently explanted. At the time of explant, there were no visible defects in the SCS system.

## 3. Discussion

This case represents a previously unreported treatment-limiting but non-life-threatening complication due to spinal cord stimulation. The onset of the patient's tinnitus and other neurologic symptoms were temporally related to activation of her system and gradually resolved within a couple of days of deactivating the therapy. Further, she had never experienced these symptoms before. In a study by Thomson et al. examining spinal cord stimulation in the 1-10 kHz range, the authors report that subjects experienced several hours to a day duration of analgesic effects of high-frequency stimulation after device deactivation [8]. Similarly, our patient also reported that her symptoms subsided over a period of two days. Another possible explanation is a psychogenic manifestation of the symptoms, which cannot be excluded even with a seemingly benign preoperative psychological assessment.

Interestingly, there are considerable similarities between some forms of tinnitus and chronic pain. Both conditions are the end results of deafferentiation of nerve signaling pathways [9–11]. In tinnitus, this leads to neuroplastic changes with a subsequent increase in background noise that ultimately leads to a “pure tone” tinnitus [12]. This pure-tone tinnitus has been suggested to be the auditory analog to chronic pain [9]. Electrical stimulation has been used as a treatment modality for tinnitus. Notably, the burst stimulation waveform was originally developed for the treatment for tinnitus via stimulation of the auditory cortex [13]. Anatomically, several recent articles have implicated the basal ganglia and the thalamus as potential generators of pure-tone tinnitus [12, 14, 15]. Additionally, fMRI studies have demonstrated activation of cortical areas of the brain with SCS. Current evidence has been lacking to demonstrate if this is a top-down or a bottom-up effect and not all waveforms induce this physiologic response [7]. Our hypothesis is that cross-talk phenomena from the afferent effects of Nevro SCS caused a central inhibition of neuronal firing which leads the patient to develop transient tinnitus. Anatomically, we hypothesized that this patient's tinnitus is localized to the basal ganglia or the thalamus.

Given this uncommon complication, a PubMed search was performed to survey the literature for reports of neurologic adverse effects from spinal cord stimulation. A case report was found of an episode of headache after placement of a low-frequency SCS for brachial plexitis. This complication was initially medically treated with eventual resolution of the adverse effects with inadvertent caudal migration of the SCS lead from C3 to C5 [16]. In addition, there have been case reports of other treatment-limiting complications such as unwanted stimulation [17], urinary retention [18], and nausea and vomiting [19]. More concerning procedural complications that have been reported include spinal nerve root or cord compression, SCS lead placement within the intrathecal space, and intraspinal placement of leads [20–22]. Additionally, there are case reports of psychiatric complications after SCS placement that resolved with explantation [23–25]. In all case reports, a low-frequency device was utilized. No case reports were found of neurological complications from “paresthesia-free” devices.

In addition to PubMed, a search was performed of the U.S. Food and Drug Administration (FDA) Manufacturer and User Facility Device Experience (MAUDE) database. The MAUDE database is a passive post-market surveillance database in which medical device companies (manufacturers), importers, and device user facilities are mandatory reporters for device-associated deaths, injuries, and malfunctions. The MAUDE database was queried for the time period of September 1st, 2015–September 30th, 2018, and the generated report was reviewed to identify all neurological adverse events, which are summarized in Table 1.

The following adverse events related to HF10 therapy were found: one patient reported instance of burning stomach pain, loss of bowel control, and dizziness with stimulation. Another patient reported onset of migraine headaches after implantation and activation for off-label treatment of headaches after a successful SCS trial. An additional patient reported the onset of headaches several months after implantation which resolved with removal of the device. There were two patients that had nausea and vomiting with stimulation that resolved with deactivation of stimulation. A patient developed oral pain 6 weeks after implant placement which resolved with cessation of stimulation and device explant. There were two patients that reported rib pain with device placement which subsequently resolved with device explantation. There was a report of a patient with CRPS type 1 and type 1 diabetes mellitus who had significant fluctuations in blood glucose with device activation and subsequent cessation of fluctuations with device deactivation. There were three reports of inappropriate shocking sensations with stimulation which resolved with removal of the device in two patients and with the cessation of the SCS trial in the third patient. Another patient reported that after 6 months of effective therapy he began to have symptoms of a panic attack whenever he used his stimulator with subsequent resolution of his symptoms with deactivation of his device. There were four reports of patients experiencing shocking sensations at their IPG sites with active stimulation. Yet another patient had new onset of chest, flank, and leg pain with stimulation which resolved with device deactivation. He had previously been receiving excellent pain relief from his device.

With respect to other SCS devices, our review of the MAUDE database over the same time period did demonstrate instances of the St. Jude Medical (now Abbott, Chicago, IL) Eon Mini having amplitude-dependent sensitivity around implant pocket with increasing stimulation. There were also several reports of shocks at the IPG site while using the stimulator. For the St. Jude Protégé series, there was a report of a stimulator increasing in stimulation intensity while near cash registers and security sensors. Two patients reported shocking sensations at the IPG site while using their device. Another patient reported intermittent shocking and tightness sensation across his chest with his stimulator. The St. Jude Prodigy had a report of a patient experiencing uncontrolled limb movement while undergoing an MRI, whereas another patient experienced uncomfortable abdominal stimulation. The St. Jude Proclaim system likewise has had reports of overstimulation and discomfort at IPG site with stimulation activation.

A major limitation of the MAUDE database is the potential for reporting bias and incomplete rate of reporting of adverse events. Thus, the true incidence of these various complications cannot be reliably deduced from its data. To highlight these differences between September 1st, 2015, and September 30th, 2018, 903 adverse events were reported regarding the Nevro Senza HF10 device, 1213 for the St. Jude Eon Mini, 174 for the St. Jude Protégé, 63 for the St. Jude Proclaim, 32 for the St. Jude Prodigy, 17 for the Medtronic (Minneapolis, MN) Restore, 1 for the Medtonic Restore Sensor, 3 for the Boston Scientific (Marlborough, MA) Precision, and 2 for the Boston Scientific Spectra systems. These findings are summarized in Table 2. It can be inferred that each of these four major SCS device companies had different reporting practices given the significant discrepancy in number of reports between them. Additional limitations of this data include inconsistencies in data entry which may further skew analysis of the data.

## 4. Conclusion

The significance of this case report and subsequent literature and FDA MAUDE database reviews is to explore less common neurologic complications of SCS therapy. While there has been significant literature on the incidence of biological complications of SCS therapy such as surgical site infection, there exist no studies on the incidence of neurological treatment-limiting complications of SCS. Further research and investigation in this area are needed so that clinicians and patients may have more complete knowledge and understanding of the potential treatment-limiting complications of spinal cord stimulation. This knowledge would help guide patients to the treatments that are likely to provide them the most favorable outcome.

## Figures and Tables

**Table 1:** Neurological complications noted in the FDA MAUDE database according to device manufacturer and number of events reported.

Device Name	Adverse neurologic event	Number of events
Senza HF10 (Nevro)	New onset of migraine	1
	New onset panic attacks	1
	New onset of chest, flank, and leg pain	1
	New onset of headache, nausea, and vomiting	2
	Oral pain	1
	Rib pain	2
	Significant blood glucose fluctuations	1
	Inappropriate shocking sensations	3
	Shocking sensation at IPG site	4

Eon Mini (St. Jude)	Amplitude-dependent sensitivity around implant pocket	1
	Shocking sensation at IPG site	7

Protégé (St. Jude)	Increase in stimulation near registers and security sensors	1
	Uncontrolled limb movement while undergoing an MRI	1
	Shocking & tightness across the chest	1

Proclaim (St. Jude)	Overstimulation and discomfort at IPG site with stimulation	1

**Table 2:** Total number of adverse events reported to FDA MAUDE database by device.

Nevro Senza HF10	903
St. Jude Eon Mini	1213
St. Jude Protégé	174
St. Jude Proclaim	63
St Jude Prodigy	32
Medtronic Restore	17
Medtronic Restore Sensor	1
Boston Scientific Spectra	2
Boston Scientific Precision	3
